# HIV-1 transcriptional silencing caused by TRIM22 inhibition of Sp1 binding to the viral promoter

**DOI:** 10.1186/s12977-015-0230-0

**Published:** 2015-12-18

**Authors:** Filippo Turrini, Sara Marelli, Anna Kajaste-Rudnitski, Marina Lusic, Carine Van Lint, Atze T. Das, Alex Harwig, Ben Berkhout, Elisa Vicenzi

**Affiliations:** Viral Pathogens and Biosafety Unit, Division of Immunology, Transplantation and Infectious Diseases, San Raffaele Scientific Institute, P2-P3 Laboratories, DIBIT, Via Olgettina n.58, 20132 Milan, Italy; Department of Infectious Diseases, Integrative Virology, University Hospital Heidelberg and German Center for Infection Research, Heidelberg, Germany; Service of Molecular Virology, Department of Molecular Biology, Université Libre de Bruxelles (ULB), Gosselies, Belgium; Laboratory of Experimental Virology, Department of Medical Microbiology, Center for Infection and Immunity Amsterdam (CINIMA), Academic Medical Center, University of Amsterdam, Amsterdam, The Netherlands; San Raffaele Telethon Institute for Gene Therapy (TIGET), San Raffaele Scientific Institute, 20132 Milan, Italy; Viral Oncology Unit, UCL Cancer Institute, London, UK

**Keywords:** HIV-1 promoter, TRIM22, Sp1-driven transcription

## Abstract

**Background:**

Intracellular defense proteins, also referred to as restriction factors, are capable of interfering with different steps of the viral life cycle. Among these, we have shown that Tripartite motif 22 (TRIM22) suppresses basal as well as phorbol ester-induced HIV-1 long terminal repeat (LTR)-mediated transcription, independently of its E3 ubiquitin ligase activity, nuclear factor kappa-light-chain-enhancer of activated B cells (NF-kB) binding to the U3 region and Tat interaction with the TAR region of the HIV-1 LTR. As basal HIV-1 transcription is driven by the transcription factor specificity protein 1 (Sp1), we have investigated whether TRIM22 could interfere with Sp1-driven transcriptional activation of the HIV-1 LTR.

**Findings:**

293T cells, devoid of endogenous TRIM22 expression, were transfected with a TRIM22-expressing plasmid together with reporter plasmids driven by the HIV-1 LTR promoter either containing or lacking Sp1 binding sites or with reporter plasmids driven by non-viral promoter sequences either containing or lacking the three Sp1 binding sites from the HIV-1 LTR. These reporter assays showed that TRIM22 efficiently inhibited Sp1-driven transcription. Knocking down TRIM22 expression in the CD4^+^ SupT1 T cell line increased the replication of Sp1-dependent HIV-1 variants. TRIM22 did not interact with Sp1, but prevented binding of Sp1 to the HIV-1 promoter, as demonstrated in protein-DNA pull down and chromatin immunoprecipitation assays.

**Conclusion:**

TRIM22 acts as a suppressor of basal HIV-1 LTR-driven transcription by preventing Sp1 binding to the HIV-1 promoter.

## Findings

Tripartite Motif (TRIM) proteins form a large family that encompasses several members with broad antiviral activities against both DNA and RNA viruses [1, 2]. TRIM22 has been previously shown to inhibit the replication of HIV-1 [3, 4], Influenza A virus [5], Hepatitis B and C viruses [6, 7] and encephalomyocarditis virus [8], although by different mechanisms. We have shown that TRIM22 inhibited both basal and PMA (phorbol, 12-myristate, 13-acetate) plus ionomycin-induced HIV-1 transcription, independently of its E3 ubiquitin-ligase activity. Furthermore, TRIM22 did not affect either NF-kB or Tat-activated HIV-1 transcription [4]. As HIV-1 basal transcription is mainly driven by the transcription factor Sp1 that binds to the three binding sites present in the core enhancer of the U3 region in the HIV-1 LTR [9], we tested whether TRIM22 interfered with Sp1-dependent transcription of HIV-1. For this purpose, Luciferase (Luc) -based reporter constructs driven by a minimal HIV-1 LTR (HIV-1 LTR Luc) containing the TATA box, Tat-binding TAR sequences, and the three Sp1 sites (WT) were transfected in 293T cells, which are devoid of endogenous TRIM22 expression. To determine whether TRIM22 inhibition of Sp1-dependent transcription was also related to the hierarchical clustering of the three Sp1 binding sites, HIV-1 LTR Luc deletion/mutation variants that retained two (ΔSp1-III), one (ΔSp1-III + II) and no Sp1 sites (mSp1) were tested in the presence and absence of TRIM22 expression. As these reporters contain two tet operator (tetO) sites for the binding of the doxycycline-inducible transcriptional activator rtTA [10], an rtTA-expressing plasmid was co-transfected in 293T cells and transcription was activated by doxycycline added to the culture medium. The effect of TRIM22 was determined by co-transfecting 293T cells with either a TRIM22-expressing plasmid or the empty control plasmid pcDNA3.1(+).

**Fig. 1:**
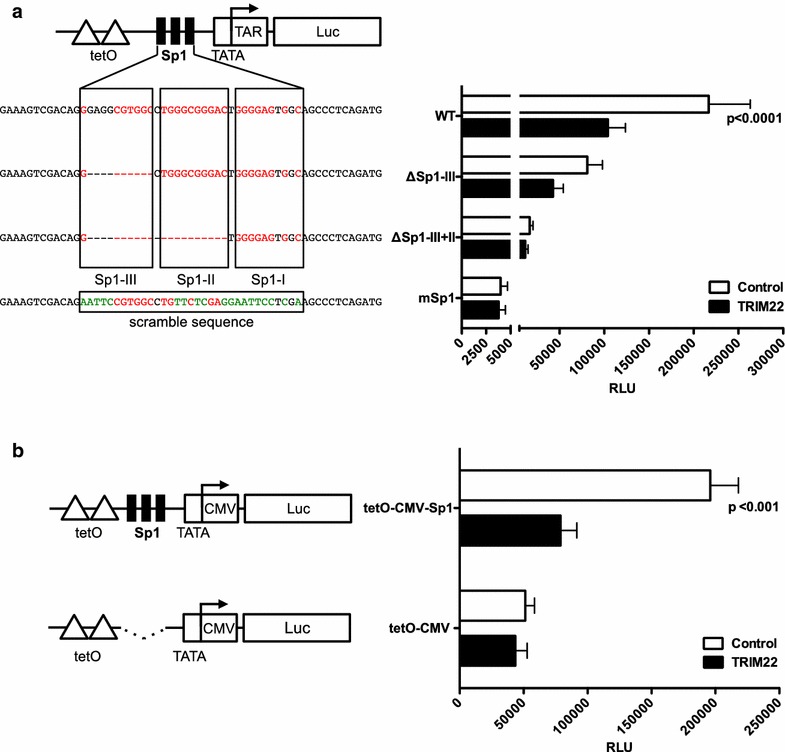
TRIM22 inhibits Sp1-driven transcription. **a** 293T cells were seeded at 2.5 × 10^5^ cells/ml in 96-well plates. 24 h post-seeding, 0.5 ng of the minimal LTR tetO Luc reporter (WT), two deletion mutants containing either two (ΔSp1-III) or one (ΔSp1-III+II) Sp1 binding sites and a mutant with a scrambled Sp1 sequence were co-transfected in 293T cells together with rtTA-V10 encoding plasmid (0.01 ng) [11] and either TRIM22-expressing or empty control pcDNA3.1+ plasmids (5 ng). Transfected cells were cultured with doxycycline (1 µg/ml). Dual-Glo Luciferase System (Promega) was used to determine the Firefly Luc activity 48 h post-transfection according to the manufacturer’s instructions. The mean of five independent experiments ± SEM is reported. p values were calculated using two-ways ANOVA. **b** Either tetO-CMV or tetO-CMV + Sp1 were co-transfected in 293T cells together with a TRIM22-expressing plasmid, or a pcDNA3.1(+) plasmid as a control. rtTA-V10 encoding plasmid was co-transfected and the cells were cultured with doxycycline. Luc activity was assessed 48 h post-transfection by a Luc assay. The mean of three independent experiments ± SEM is shown. The p values were calculated using the two-ways ANOVA

In the absence of TRIM22 expression, the progressive deletion of the Sp1 binding sites significantly decreased HIV-1 transcription, whereas mutation of all three sites resulted in low but still detectable Luc levels. TRIM22 expression significantly reduced Luc activity of the WT construct with three Sp1 binding sites (2.08 fold reduction; Fig. 1a). This inhibitory effect was less significant for the ΔSp1-III (1.89 fold) and ΔSp1-III + II (1.41 fold) constructs with two and one Sp1 sites, respectively, and absent for the mSp1 construct (1.05 fold) without Sp1 sites. Thus, TRIM22 inhibited the Sp1-mediated transcription of HIV-1 LTR and this inhibition correlated with the number of Sp1 sites and all three sites appear to be required for maximal inhibition.

To verify whether TRIM22 could inhibit Sp1-mediated transcription activation out of the context of the HIV-1 LTR, a reporter construct driven by two tetO sites coupled to the minimal cytomegalovirus (CMV) promoter and lacking any HIV-1 related promoter sequence (tetO-CMV configuration) was tested in the presence or absence of TRIM22-expressing plasmid. As shown in Fig. 1b, TRIM22 expression did not affect the Luc activity driven by tetO-CMV promoter. A similar promoter construct that included the three HIV-1 Sp1 binding sites (tetO-CMV-Sp1 configuration) was also tested. The presence of Sp1 binding sites increased the Luc activity ~ fourfold and, importantly, restored the inhibitory effect of TRIM22 on promoter-dependent transcription. All together, these findings demonstrate that TRIM22 expression inhibits Sp1-driven transcription from the HIV-1 LTR.

**Fig. 2:**
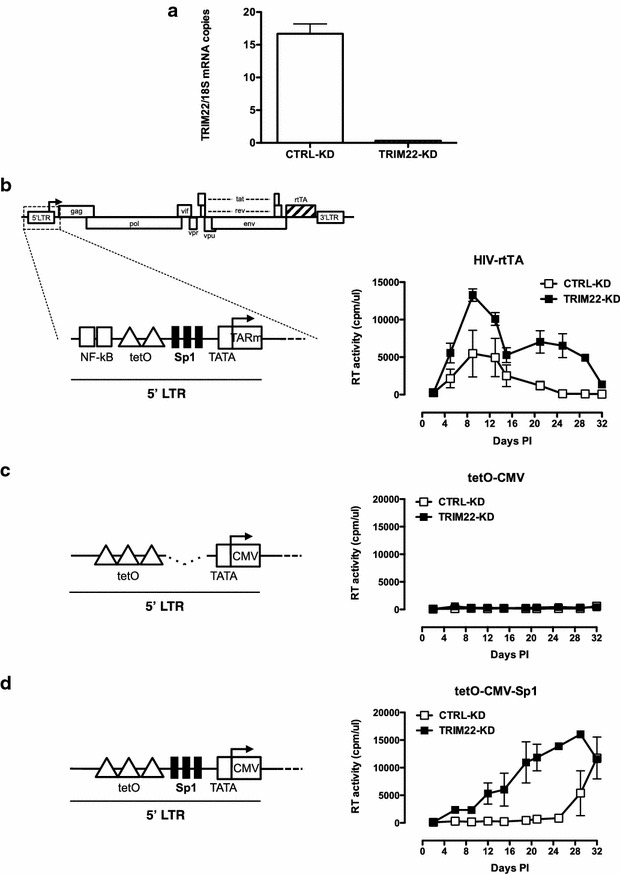
TRIM22 inhibits Sp1-driven replication. **a** SupT1 cells were transduced with either pLKO.1/TRIM22_shRNA_ (TRIM22-KD) or pLKO.1/random_shRNA_ silencing control (CTRL-KD) lentiviral vectors and selected in culture by the addition of puromycin (0.2 µM). TRIM22 expression was assessed by absolute quantitative real-time PCR and normalized on the total number of 18S mRNA copies [4]. Specificity of TRIM22 knockdown was previously assessed [5]. Replication of the wild-type (**b**), tetO-CMV (**c**) and tetO-CMV-Sp1 (**d**) HIV-rtTA virus variants in TRIM22-KD and CTRL-KD SupT1 cell lines. Virus stocks were generated by transfection of 293T cells with DNA of the infectious clone. Cells were cultured in the presence of doxycycline (1 µg/mL) and virus-containing supernatant was harvested after 48 h and tested for Mg^2+^-dependent reverse transcriptase (RT) activity assay [4] yielding measurable amounts of RT activity (~4000 cpm/µL). Viral supernatants containing 1 × 10^4^ cpm-equivalents were added to 5 × 10^5^ SupT1 TRIM22-KD or KD-control cells and spinoculated at 2900 rpm for 2 h at 37 °C. Cells were cultured at 5 × 10^5^ cell/well in duplicate in the presence of doxycycline (1 µg/mL). Kinetics of viral replication were measured by RT activity assay in the supernatant collected every 3–4 days post-infection (PI) up to 32 days. Mean ± SEM of three independent infections in triplicates are shown

Next, we assessed whether TRIM22 inhibition of Sp1-driven transcription could be observed in the context of full-length replication-competent HIV-1. We took advantage of different HIV-rtTA infectious molecular clones that use the incorporated tetracycline-controlled (Tet-On) gene expression system for activation of transcription and that allow replication with alternative promoter configurations [10, 12]. In the “wild-type” HIV-rtTA strain, the Tat/TAR transcription mechanism was inactivated through mutation of TAR, the rtTA gene was inserted at the site of the *nef* gene and tetO elements were inserted between the NF-kB and Sp1 sites in the U3 promoter region. To test whether TRIM22 targeted Sp1, we included two variants with either the tetO-CMV or tetO-CMV-Sp1 promoter configuration [11]. Viral stocks were generated by transfecting 293T cells with the DNA of the three infectious clones and virion production was quantified by measuring the reverse transcriptase (RT) activity. Equal amounts of RT activity were used to infect human CD4^+^ SupT1 cells that had been transduced with a lentiviral vector expressing a shRNA against TRIM22 (TRIM22-KD cells) or with a non-silencing control vector (CTRL-KD cells). As shown in Fig. 2a, transduction with the shRNA-TRIM22 vector efficiently knocked down TRIM22 RNA expression. Upon infection of the TRIM22-KD and CTRL-KD SupT1 cells with the different HIV-rtTA variants, virus replication was followed up to 32 days post-infection (PI).

HIV-rtTA replicated more efficiently in TRIM22-KD cells than in CTRL-KD cells (Fig. 2b). In this virus, three Sp1 sites are present in the U3 promoter region, which explains why TRIM22 negatively influences viral replication. The tetO-CMV virus did not show any replication upon infection of CTRL-KD and TRIM22-KD SupT1 cells, which is likely due to the absence of NF-kB and Sp1 binding sites (Fig. 2c). The tetO-CMV-Sp1 virus replicated also very poorly in CTRL-KD cells (RT activity became detectable only from day 29 PI), but it replicated significantly better in the TRIM22-KD SupT1 cells (Fig. 2d). Altogether, these results demonstrate that TRIM22 interferes with HIV-1 replication that is dependent on Sp1 binding sites in the LTR.

**Fig. 3:**
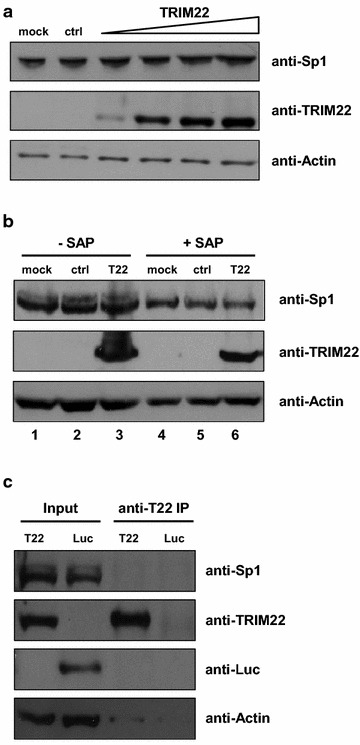
TRIM22 does not interact with Sp1 and does not alter Sp1 expression and phosphorylation. **a** 293T cells were transfected with 1.5, 3, 4.5, or 6 µg of a TRIM22-expressing plasmid, equalizing the DNA doses with empty pcDNA3.1(+) vector. 48 h post-transfection, whole cell extract (WCE) was analyzed by western blotting with anti-Sp1, anti-TRIM22 and anti-Actin antibodies (Abs). A similar level of Sp1 was observed in the mock-treated cells, control cells (empty pcDNA3.1 only) and cells expressing increasing concentration of TRIM22 expressing plasmid. One representative of three independent experiments is shown. **b** 293T cells were transfected with 6 µg of either a TRIM22-expressing plasmid (T22) or pcDNA3.1(+) plasmid (ctrl). 48 h post-transfection, WCE was prepared in the presence of PhosStop phosphatase inhibitor cocktail (Roche). An aliquot of 50 µg was treated with 5 I.U. of SAP (Roche) for 45 min at 37 °C and subjected to western blot analysis. The level of phosphorylated Sp1 was not altered by TRIM22 expression (*lanes 2* and *3*). SAP treatment caused the disappearance of the phosphorylated forms of Sp1 (*upper band*), without affecting overall Sp1 levels detected between TRIM22-expressing and control conditions (*lanes* *5* and* 6*). One representative of two independent experiments is shown. **c** 293T cells were transfected with either TRIM22-expressing or Luc-expressing plasmid as unrelated control protein. 24 h post-transfection, 90 % of WCE was immunoprecipitated 2 h at 4 °C with anti-TRIM22 (Abnova) Ab, whereas a 10 % volume was saved as input fraction. Immunocomplexes were captured using magnetic Dynabeads protein G (Life Technologies), according to the manufacturer’s protocol. Western blotting was performed with anti-Sp1 (Millipore), anti-Firefly Luciferase (Millipore) and anti-Actin Abs. Endogenous Sp1 did not immunoprecipitate with TRIM22. One representative of two independent experiments is shown

As TRIM22 is an E3 ubiquitin ligase [8] and poly-ubiquitination targets Sp1 to proteasome-dependent degradation [13], we investigated whether TRIM22 expression resulted in the degradation of Sp1. However, Sp1 expression was not altered by TRIM22 transfection (Fig. 3a), which is consistent with our previous observation that TRIM22 inhibition of HIV-1 transcription is independent of its E3 ubiquitin ligase [4] and indicates that TRIM22 does not promote Sp1 degradation. Then we evaluated whether an alteration of Sp1 phosphorylation, known to regulate Sp1-dependent transcriptional activity [14], could explain TRIM22 inhibition of Sp1-driven transcription. As shown in Fig. 3b, the level of phosphorylated Sp1 was not altered by TRIM22 expression (lanes 2 and 3). Shrimp Alkaline Phosphatase (SAP) treatment caused the disappearance of the phosphorylated forms of Sp1 (upper band), without affecting overall Sp1 levels detected between TRIM22-overexpressing and control conditions (lanes 5 and 6). The analysis of nuclear extracts prepared in the absence or presence of SAP by two-dimensional protein gel electrophoresis confirmed that TRIM22 did not cause an alteration of Sp1 phosphorylation state (data not shown). Furthermore, co-immunoprecipitation (co-IP) experiments showed that endogenous Sp1 did not co-precipitate with TRIM22 in 293T cells transfected with a TRIM22 expressing plasmid (Fig. 3c) suggesting a lack of interaction between the two proteins.

**Fig. 4:**
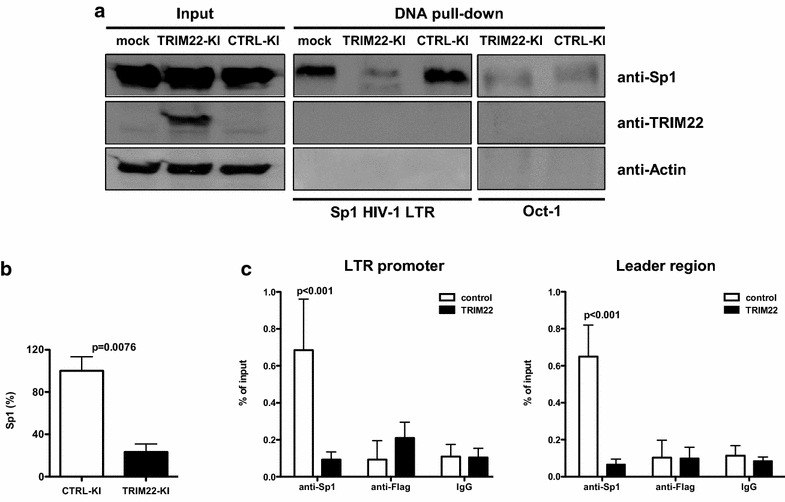
TRIM22 prevents Sp1 binding to the HIV-1 promoter. **a** 293T cells were transduced with either pAIP/TRIM22 (TRIM22-KI) or pAIP/empty (CTRL-KI) lentiviral vectors [4] and selected in culture by the addition of puromycin (0.5 µM). WCE from 50 × 10^6^ mock-transduced, CTRL-KI and TRIM22-KI cells was incubated overnight at 4 °C with 40 pmol of 5′-biotinilated double-stranded DNA probes containing either the three Sp1 binding sites of the HIV-1 LTR (as shown in Fig. 1a) or the consensus site of Oct-1 (5’-TGTCGAATGCAAATCACTAGAA-3’). Each sample was pulled-down using streptavidin-conjugated beads (Life Technologies) and analyzed by western blot in parallel with the input fraction (1 % of the pull-down) using anti-TRIM22 (Sigma-Aldrich), anti-Sp1 and anti-Actin (Santa Cruz Biotechnologies) Abs. One representative of three independent experiments is shown. **b** The Sp1 band intensity of the input and LTR-pull-down fractions from either CTRL-KI or TRIM22-KI cells was quantified by ImageJ. The mean ± SD of three independent experiments is reported. p value was calculated using a paired *T* test. **c** Modified pREP10 episomal vector containing the Luc reporter driven by the HIV-1 LTR [15] was co-transfected in 1.2 × 10^8^ 293T cells with Flag-TRIM22-expressing plasmid (provided by Dr. Nadir Mechti, Montpellier, France [8]) or empty plasmid. 48 h post-transfection, proteins were crosslinked to the DNA by formaldehyde incubation. Cell extracts were sonicated to shear chromatin obtaining an average fragment size ranging from 50 to 400 base pairs. IP was performed by using anti-Flag (Sigma-Aldrich), anti-Sp1 and anti-IgG (Santa Cruz Biotechnologies) Abs. Both input (1/1000) and immunoprecipitated DNAs were reverse-crosslinked, purified from proteins and quantified by two real-time SYBR-Green qPCR (LTR promoter and leader region). LTR promoter primer sequences: for 5′-GACTTTCCGCTGGGGACTTTC-3′, rev 5′-CTAACCAGAGAGACCCAGTAC-3′. Leader region primer sequences: for 5′-TGGAAAATCTCTAGCAGTGGC-3′, rev 5′-GAGTCCTGCGTCGAGAGATCT-3′. The amount of immunoprecipitated DNA was normalized to the input DNA and expressed as percentage on input fraction. The mean ± SEM of 3 independent experiments is shown. The p value was determined by the two-way ANOVA

We next tested whether TRIM22 influenced the in vitro binding of Sp1 to the DNA binding sites as present in the HIV-1 LTR. For this purpose, a 293T cell line stably expressing TRIM22 (TRIM22-KI) and its control (2CTRL-KI) were generated by lentiviral transduction. As expected, TRIM22-KI or CTRL-KI cells expressed similar level of Sp1 as compared with mock-transduced 293T cells (Fig. 4a). However, when incubated with a DNA probe containing the three Sp1 binding sequences of the HIV-1 LTR, the whole cell extract of TRIM22-KI cells either prepared in the absence (Fig. 4b) or in the presence (not shown) of phosphatase inhibitors showed a significant reduction (~77 %) of Sp1 binding as compared with control cells. In contrast, the binding of the unrelated constitutively expressed transcription factor Oct-1 to its consensus sequence was not altered by TRIM22 expression. These findings demonstrate that TRIM22 specifically reduces the binding of Sp1 to its target DNA sequences in vitro.

Next, we performed chromatin IP (ChIP) on 293T cells transfected with an HIV-1 LTR-Luc plasmid in the presence or absence of a Flag-TRIM22 expression plasmid to further analyze the impact of TRIM22 on Sp1 binding to the viral promoter in the context of the complete HIV-1 LTR in vivo. IP was performed using anti-Sp1 and anti-Flag Abs to isolate Sp1-bound and TRIM22-bound DNA, respectively. The DNA released from the immune-complexes was analyzed by two independent quantitative real-time PCR that generated amplicons between −103/+14 (LTR promoter [15]) and +164/+244 (leader region [16]), respectively, relative to the transcription start site (+1). The LTR promoter PCR encompasses the three Sp1 binding sites whereas the leader region PCR is nearby two Sp1 binding sites. The rate of amplification was normalized on cross-linked non-IP chromatin (% of input). As expected, analysis of the Sp1-IP samples revealed reduced binding of the HIV-1 DNA in the presence of TRIM22, confirming that TRIM22 interferes with Sp1 binding to the HIV-1 LTR (Fig. 4c). In contrast, when the anti-Flag Ab was used to IP TRIM22 or when unrelated immunoglobulins (IgGs) were used as negative control, a similar low level of LTR fragments was detected in the presence and absence of TRIM22.

Overall, these results demonstrate that TRIM22 causes transcriptional repression of HIV-1 by interfering with the binding of Sp1 to the HIV-1 LTR promoter region. Consistent with the lack of DNA binding domains in TRIM proteins [17], we did not observe direct binding of TRIM22 to the HIV-1 LTR. These observations together with our inability to co-IP TRIM22 and Sp1 suggest an indirect effect of TRIM22 on Sp1 binding to the HIV-1 LTR, which may involve one or several other factors. This/these unidentified TRIM22 partner(s) could either promote a transcriptionally silenced heterochromatin configuration, as in the case of COUP-TF interacting protein 2 (CTIP2) [15] and c-Myc [18], or stimulate post-translational modifications of Sp1, as previously suggested for histone deacetylases (HDAC) [19] and p300 [20], thereby affecting its DNA-binding affinity. Furthermore, by preventing Sp1 binding to the promoter, TRIM22 might favor binding of Sp3, another Sp family member considered a repressor of transcription [21].

Sp1 is an ubiquitous factor and TRIM22 is expressed in different immune cells and induced by type I interferons [22]. Therefore, TRIM22 has the potential to inhibit the transcription of several host genes driven by Sp1 and to suppress cellular or even tumor growth [23]. Recent work has highlighted the peculiar configuration of the three adjacent Sp1 binding sites of the HIV-1 promoter as a guanine-rich sequence that can fold into a G-quadruplex (G4) structure [24] previously identified in eukaryotic oncogenes [25]. Pharmacological stabilization of this structure promotes transcription silencing of both the HIV-1 LTR [24] and oncogenes [25]. By preventing Sp1 binding, TRIM22 could favor the formation of the G4 structure in the HIV-1 LTR but also in oncogenes.

In conclusion, in the present study we demonstrate that TRIM22 acts as a negative regulator of HIV-1 replication via inhibition of basal Sp1-driven proviral transcription. We can thus speculate that TRIM22 favors either the establishment or maintenance of HIV-1 latency. TRIM22 could therefore represent a novel target for pharmacological interference in latently infected cells harboring replication-competent proviruses.
